# Quantitative analyses of squamate dentition demonstrate novel morphological patterns

**DOI:** 10.1371/journal.pone.0257427

**Published:** 2021-09-10

**Authors:** Kiana Christensen, Keegan M. Melstrom

**Affiliations:** 1 Natural History Museum of Utah, University of Utah, Salt Lake City, Utah, United States of America; 2 Natural History Museum of Los Angeles County, Los Angeles, California, United States of America; Ecole Normale Supérieure de Lyon, FRANCE

## Abstract

Squamates are ideal subjects for investigating relationships between diet and dental patterns because they exhibit wide dietary diversity, marked variation in dental shape, and are taxonomically abundant. Despite this, well-established links between diet and dental morphology are primarily qualitative in nature, with specific patterns of squamate dental complexity remaining largely unknown. Here, we use quantitative methods and a broad taxonomic dataset to quantify key patterns in squamate dental morphology, including re-examining the relationship between dentition and diet, testing for differences in complexity between dentigerous elements, and exploring the effect of ontogenetic dietary shifts in dental complexity in two iguanid genera. Our findings support previous research by demonstrating that species consuming more plant material possess more complex teeth. We did not find significant complexity differences between the left and right dentigerous elements nor the upper and lower jaws, with the exception of *Amblyrhynchus cristatus*, the marine iguana, which possesses significantly more complex dentary teeth than premaxillary and maxillary teeth. We find discordant patterns when testing for dental complexity changes through ontogeny. *Amblyrhynchus*, which is primarily herbivorous throughout its lifetime, increases dental complexity through ontogeny, whereas *Ctenosaura*, which is generally insectivorous as juveniles and herbivorous as adults, decreases dental complexity. Although preliminary, this research documents and quantifies novel patterns of squamate dental complexity and exhibits the possibilities for further research on the diversity of squamate dental morphology.

## Introduction

Despite their dietary variation, differences in morphology, and taxonomic diversity, studies exploring patterns between tooth shape and diet are remarkably rare for squamates. Key patterns related to diet in iguanids reveal that herbivorous taxa have sharp, blade-like, cuspate teeth; insectivorous taxa have blunted, stout, non-cuspate teeth; and predaceous species have stout, conical teeth with variable cuspation [1] although other authors focused on additional groups including agamids and macroteiids [2–5]. These qualitative descriptions are beneficial for describing prominent tooth characteristics and comparing dentition based on shared features but may lack repeatability due to authors characterizing traits differently.

Quantitative methods are becoming increasingly common for examining mammalian dentitions but less so in squamates [6–11]. These methods are advantageous because they are repeatable and allow for direct comparisons of different dentitions without the use of homologous landmarks. In particular, dental complexity quantifies phenotypic tooth morphology of the occlusal surface and has recently been used to investigate the relationship between squamate diet and tooth complexity [11]. Melstrom [11] examined a wide range of saurians with a variety of diets and demonstrated that taxa within the same dietary group have similar dental complexities [11]. Generally, sampled individuals consuming primarily plant material have complex teeth, whereas species consuming animal material possess simpler teeth. These trends support previous qualitative and quantitative literature by showing that species with similar diets have similar dental morphology and dental complexities [1, 2, 6].

Beyond this broad pattern, more detailed trends in squamate dental complexity remain only superficially studied. For example, in many squamates small sharp, conical anterior teeth gradually transition to larger, blunter, multi-cusped posterior teeth in a range of heterodont taxa [2]. Additionally, studies suggest that the upper tooth row appears to be a mirror image of the lower tooth row and the left and right sides of the mouth are identical [1, 2]. Previous research has suggested several asymmetrical traits in reptiles [12], this work, however, has not quantitatively demonstrated that upper and lower tooth rows have similar complexities, nor has it determined that left and right dentigerous elements have similar complexities.

Finally, previous research documented many squamate taxa undergo a dietary shift from insectivorous or carnivorous juveniles to herbivorous adults [13–16]. In some species that undergo a shift in diet, studies have shown that new molariform teeth become progressively larger as individuals become older [4, 17]. Additionally, changes in dental shape and morphology throughout an individual’s lifetime have been linked to dietary shifts [18].

Here, we seek to expand the use of quantitative metrics on squamate dentitions to preliminarily document key patterns. In particular, we examine differences in dentigerous elements, use the relationship between diet and dental complexity to hypothesize the diet of living taxa, and investigate dental complexity changes in two iguanids that undergo an ontogenetic shift in diet. We hypothesize that results from this study will reflect those of Melstrom [11], demonstrating that herbivores have more complex teeth than carnivores. Furthermore, we predict complexities of the upper and lower tooth row will be similar and the complexities of the left and right sides of the mouth will be nearly identical. Lastly, we hypothesize that dental complexity will increase in taxa transitioning from insectivorous juveniles to herbivorous adults. We hope to expand quantitative measurements of dental complexity and recognize new patterns to ultimately spur future research.

## Materials and methods

We investigated the dentition of 17 species from 14 genera (six specimens were not identified to species level). We molded the premaxillae, maxillae, and dentaries of 31 specimens in museum collections whereas eight specimens were downloaded from MorphoSource (*A*. *cristitellus*, *C*. *namakuiyus*, *D*. *blanfordii*, *H*. *horridum*, *L*. *borneensis*, *S*. *crocodilurus*, *V*. *prasinus*, and *X*. *rectocollaris)*. Scans of our casts are available on MorphoSource by searching for manuscript title or author. Dentitions were molded using Reprosil light body catalyst and base molding material and subsequently cast with EPO-TEK 301 epoxy resin, which has previously been shown to capture sub-micron details [19]. The paleontology collection of the Natural History Museum of Utah reposits the molds and casts. Iguanids are best represented in this study because their diets are well-documented and several taxa undergo clear dietary shifts through ontogeny [13–15, 20]. The specimens obtained from MorphoSource exhibit a range of diets, dental morphologies, and were sufficiently high-resolution to capture dental details.

Ontogenetic maturity of sampled individuals ranges from juvenile to adult. The design of the current study is cross-sectional rather than longitudinal, meaning that individuals of different ages were examined at one point in time, rather than following one individual throughout its lifetime. Relative ages of specimens can be estimated by snout-vent length (SVL) which increases with age [21]. Given this correlation, we can estimate relative age by measuring their skull length, with smaller specimens inferred to be juveniles whereas larger individuals are adults.

### Diet classification

We classify specimens in this study into four dietary categories: herbivores, omnivores, insectivores, and carnivores. We gathered information on each species’ diet from the literature [20, 22–24]. Previous authors obtained data regarding the percentage of plant or animal material consumed in several ways, including volumetric and mass measurements of gut contents and occurrence and type of food items eaten [24]. Diet classification in this study follows guidelines set forth by Cooper and Vitt [24] who proposed that carnivores consume over 90 percent animal material and less than 10 percent plant material, whereas herbivores consume over 90 percent plant material and less than 10 percent animal material. Insectivores follow the same guidelines as carnivores but are distinguished by the fact that the animal material they consume is mostly invertebrates, specifically arthropods, rather than vertebrates. Omnivores eat a mixture of plant and animal material, and are thus classified by consuming between 10 and 90 percent plant material. Unfortunately, some taxa do not have sufficient dietary information. The diet of several species has not yet been studied or confidently determined (*Hydrosaurus amboinensis*, *Lanthanotus borneensis*), and other species’ diets have only been described qualitatively [1, 24, 25]. For the latter group, diet was determined based on a consensus of available data.

Several squamate species included in the current study have stable diets throughout their lifetime, whereas other species undergo a shift in diet from juveniles to adults [13, 15, 16, 20]. Both *Iguana iguana* and *Dipsosaurus dorsalis* are herbivorous throughout their lifetimes [13, 14, 23, 26, 27], whereas *Ctenosaura pectinata* and *Ctenosaura similis* undergo an ontogenetic shift in diet from insectivore to herbivore [13, 20, 23]. Studies document that adult *Amblyrhynchus cristatus* eats primarily plant material, yet hatchlings possess a greater percentage of prey in their diet as compared to adults [15, 24].

### Dental complexity measurement and analysis

We investigated the dental complexities of four elements: right premaxilla and maxilla, left premaxilla and maxilla, right dentary, and left dentary. We chose to compare dentigerous elements rather than mesial to distal because although several species show distinct anterior and posterior teeth, many taxa demonstrate a complexity gradation from simple anterior teeth to complex posterior teeth, and other species are homodont and therefore do not have a morphologic difference between anterior and posterior so determining a dividing line between anterior and posterior would be inconsistent. By comparing a single element, we have a consistent set of teeth that can be compared between species, and we can calculate a complexity value that includes both anterior and posterior teeth, regardless of the degree of heterodonty. In some cases, specimens were missing one or more elements, which resulted in differences in dataset sizes. Casts were scanned at the University of Utah Small Animal Imaging Core Facility using a Siemens INVEON microCT scanner with a voxel resolution of 35 microns. Information on scanning procedures of MorphoSource specimens is available on their webpages. Scans were edited to remove unnecessary elements including extraneous bone and additional casting material. The final scans were oriented to show only the occlusal surface of all tooth crowns in an element.

We calculated dental complexity using the programs Surfer 8 (Golden Software) for Windows and Surfer Manipulator [6]. Surfer 8 is a 3D geographic information system program that uses spatial information such as coordinates and orientation to analyze 3D objects’ patterns and features, in this case squamate dentition. Surfer Manipulator was then used to calculate dental complexity values. Orientation patch count rotated (OPCR) quantifies tooth complexity by grouping neighboring pixels with the same orientation (e.g., North, South, etc.) into a patch, then summing the number of patches of the entire occlusal surface of the tooth row [6]. A greater number of patches on the occlusal surface indicates a more complex element, whereas a lower number of patches indicates a simpler element. The files were saved with four different levels of resolution: 150 data rows per element, and 25, 40, and 50 data rows per tooth (RPT). OPCR data presented here were analyzed using values calculated at a resolution of 25 RPT. This resolution was used to avoid issues related to scan artifacts and allows for the combination of data from multiple CT sources (e.g., MorphoSource and microCT scans) [11].

Squamates undergo regular tooth replacement, which resulted in most individuals missing one or several teeth at the time dentitions were scanned [28, 29]. To account for disparities in tooth number between elements, the number of teeth per element was recorded and the average OPCR-value per tooth was calculated for individual elements, upper and lower tooth rows, and the entire dentition. The average OPCR per tooth of an individual element was determined by dividing the total OPCR-value of an element by the number of teeth measured. The average OPCR per tooth of the dentary was calculated by summing the left and right dentary then dividing by the number of dentary teeth. The same calculation was used for the premaxilla and maxilla. The average OPCR per tooth of an individual’s entire dentition (avgOPCR) was calculated by summing the OPCR-values of each element and dividing it by the total number of teeth.

### Statistical analysis

We completed all statistical tests using the PAST3 computer software [30]. We performed a Kruskal–Wallis test to assess significant differences between the medians of each dietary category, whereas we determined pairwise differences between dietary categories and elements using a Mann–Whitney *U* test. To examine the correlation between skull length and average OPCR, we used a linear regression model. We used this model for species that had over five individuals in order to only present trends with sufficient data. For ontogenetic trends of the genus *Ctenosaura*, phylogenetic relationships were not taken into account because taxa were closely related.

## Results

### Relationship between diet and dentition

**Fig 1:**
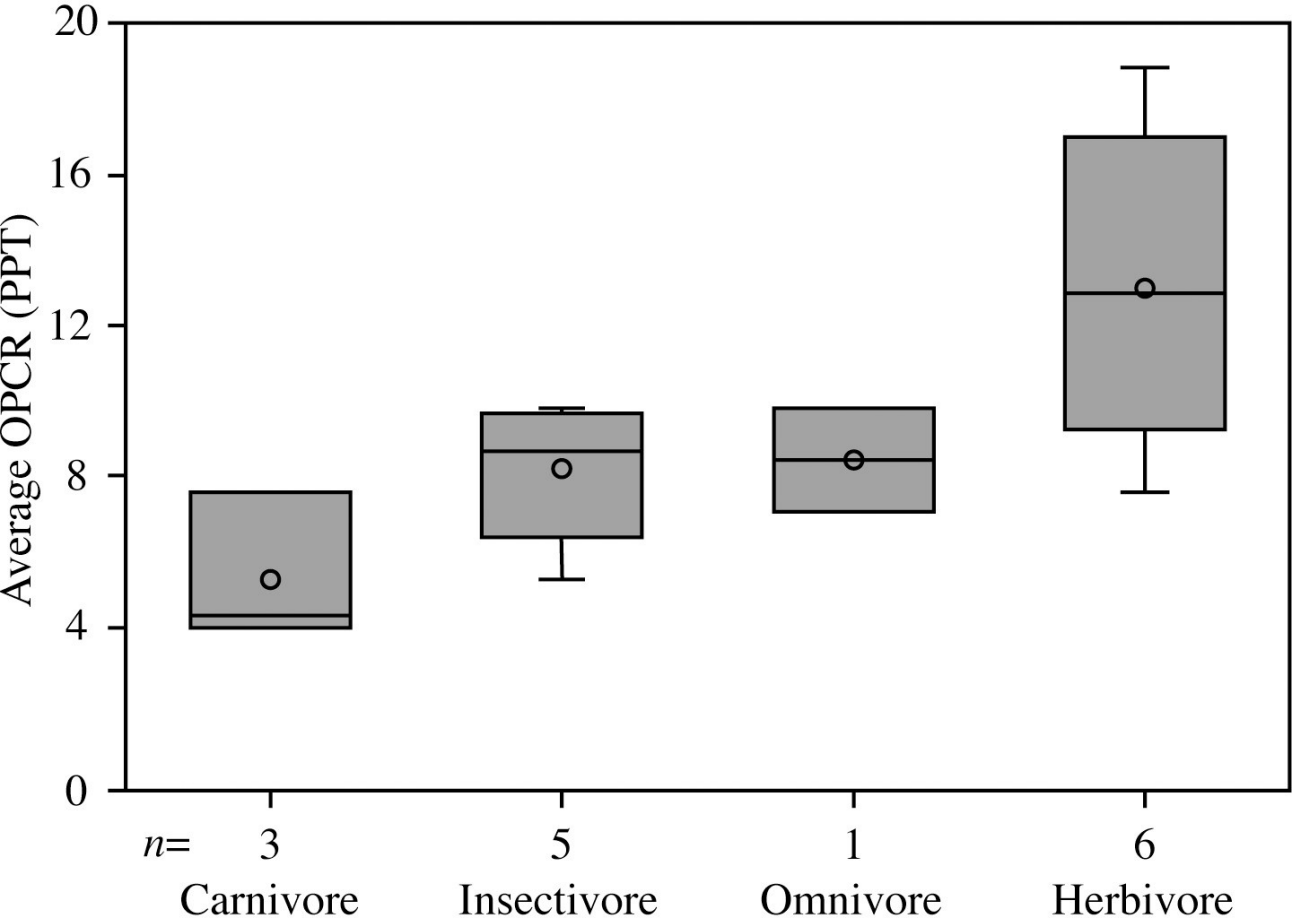
Calculated range of average dental complexities for four dietary groups. The median is represented by a horizontal line within each box and the mean is designated by the small circle. Boxes encompass the exclusive median. The range is represented by the upper and lower short horizontal lines. Individuals with an unknown diet or unknown species identification are not included. *n* represents the number of species included in each dietary category.

Species exhibit a wide range of diets and dental complexities (Table 1). Individuals within a single dietary group show similar dental complexities to each other and different complexities compared to individuals of other dietary groups (Table 2). Species within each diet category show a variety of dentitions and range from homodont to varying degrees of heterodonty. Dental complexities of each dietary group reflect patterns observed in previous research [11]. Complex dentitions generally correspond to a high percentage of plant material in the diet, whereas low dental complexity corresponds to a greater degree of animal material (i.e., vertebrate or invertebrate). Herbivores demonstrate the highest complexity, followed by omnivores, insectivores, and lastly carnivores with the lowest dental complexity (Fig 1).

**Table 1:** Information of specimens used in this study including identification, avgOPCR per tooth of all included elements, diet classification, percentage of plant material in diet, total number of teeth in specimen, and elements included in OPCR calculation.

Name	Specimen no.	Avg. OPCR	Diet	% plant in diet	# of teeth	Elements included
*A*. *cristatus*	FMNH 15073	18.780	Herbivore	100	71	All
*A*. *cristatus*	FMNH 22042	18.458	Herbivore	100	52	RD, LM, RM
*A*. *cristatus*	AMNH 31700	17.129	Herbivore	100	18	LD, LM
*A*. *cristatus*	AMNH 75942	17.190	Herbivore	100	32	LD, LM
*A*. *cristatus*	AMNH 31591	17.736	Herbivore	100	32	LD, LM
*A*. *cristatus*	FMNH 15072	14.036	Herbivore	100	34	LD, LM
*A*. *cristatus*	FMNH 22100	13.485	Herbivore	100	18	LM, RM
*A*. *cristatus*	FMNH 13547	15.762	Herbivore	100	82	All
*A*. *cristatus*	FMNH 22094	18.980	Herbivore	100	71	All
*An*. *cristitellus*	UF 47686	8.6294	Insectivore	?	80	All
*B*. *vittatus*	FMNH 98363	9.7474	Insectivore	8.76	44	LD, RD
*B*. *vittatus*	FMNH 98361	9.9243	Insectivore	8.76	84	All
*Co*. *namakuiyus*	CAS 254912	6.8728	Insectivore	–	62	All
*C*. *hemilopha*	AMNH 57408	9.8898	Omnivore	86.4	57	LD, LM
*C*. *hemilopha*	AMNH R-147855	7.1916	Omnivore	86.4	15	LM
*C*. *pectinata*	AMNH R-71837	8.1309	Herbivore	100	21	LM
*C*. *similis*	FMNH 6175	8.0932	Herbivore	98	55	LM, LD, RD
*C*. *similis*	FMNH 211849	14.108	Herbivore	98	92	All
*C*. *similis*	AMNH 69627	8.7142	Herbivore	98	90	All
*C*. *similis*	AMNH 38949	11.847	Herbivore	98	27	LD
*Ctenosaura sp*.	FMNH 98370	12.115	–	–	38	LD, RD
*Ctenosaura sp*.	FMNH 22103	10.977	–	–	51	LD, RD
*Ctenosaura sp*.	FMNH 211835	8.2734	–	–	48	LD, RD
*Ctenosaura sp*.	FMNH 98371	13.250	–	–	24	LD
*D*. *dorsalis*	FMNH 249786	13.011	Herbivore	97.3	78	All
*D*. *dorsalis*	FMMH 98376	11.387	Herbivore	97.3	41	LM, RM
*D*. *dorsalis*	FMNH 98377	11.060	Herbivore	97.3	71	All
*Dr*. *blanfordii*	UF 61535	8.9768	Insectivore	–	76	All
*He*. *horridum*	UF 153328	4.3855	Carnivore	–	24	RM, LD, RD
*H*. *amboinensis*	AMNH R-140825	7.8736	?	?	34	LD, RD
*H*. *pustulatus*	FMNH 14952	10.501	Herbivore	?	43	All
*Hydrosaurus sp*.	FMNH 236131	10.131	–	–	42	All
*Iguana sp*.	FMNH 22291	8.8526	Herbivore	100	34	LM, RM
*I*. *iguana*	FMNH 51680	10.017	Herbivore	100	100	LM, LD, RD
*I*. *iguana*	FMNH 22492	7.6949	Herbivore	100	78	All
*L*. *borneensis*	UF 16268	4.2324	?	–	24	RM, LD, RD
*S*. *crocodilurus*	UF 60925	7.7160	Carnivore	–	58	All
*V*. *prasinus*	UF 71411	4.0979	Carnivore	–	42	All
*X*. *rectocollaris*	UF 51438	5.4313	Insectivore	–	78	All

Abbreviations: right premaxilla and maxilla (RM), left premaxilla and maxilla (LM), right dentary (RD), left dentary (LD), diet not found in previous literature (?), level of species unidentified so diet could not be determined (–).

**Table 2:** Statistical analysis comparing OPCR-values between dietary categories.

Diets compared	Statistical test	*P*-value	Significance
Herbivore, Insectivore, Carnivore	Kruskal–Wallace	***P* < 0.01**	Significant
Herbivore vs. Insectivore	Mann–Whitney *U*	***P* < 0.01**	Significant
Herbivore vs. Carnivore	Mann–Whitney *U*	***P* < 0.01**	Significant
Insectivore vs. Carnivore	Mann–Whitney *U*	***P* =** 0.09	Not Significant

The Kruskal–Wallace test compared multiple dietary categories, and the Mann–Whitney *U* test compared two dietary categories to each other. A *P*-value of less than .05 is considered significant (bolded). Omnivores were not compared because only two omnivorous specimens were sampled.

Six herbivorous taxa were included in the study: *Amblyrhynchus cristatus*, *Ctenosaura pectinata*, *Ctenosaura similis*, *Dipsosaurus dorsalis*, *Hydrosaurus pustulatus*, and *Iguana iguana*. Herbivores demonstrate the broadest range of dental complexities. On average, *Ctenosaura pectinata* and *Iguana iguana* show the lowest average species complexities, measuring 8.131 and 8.726 patches per tooth (PPT), respectively. The highest complexity is exhibited by *Amblyrhynchus cristatus*, with a species average OPCR-value of 17.145 PPT. The remaining species, *Ctenosaura similis*, *Dipsosaurus dorsalis*, and *Hydrosaurus pustulatus* show intermediate OPCR-values of 10.618, 11.907, and 10.502 PPT, respectively. Herbivorous lizards possess a variety of tooth morphologies; for example, *Amblyrhynchus cristatus* has tricuspid teeth throughout the entire dentition, whereas *Iguana iguana* has serrated teeth posteriorly and simple teeth anteriorly. Other herbivorous species exhibit cuspate teeth posteriorly and sharp, conical teeth anteriorly.

Only one omnivorous species is included in the study, *Ctenosaura hemilopha*, which possesses a species average OPCR-value of 8.990 PPT, a value at the lower end of herbivore complexities (8.131–17.145 PPT) and upper limits of insectivore complexities (5.431–9.865 PPT). The dental morphology of *Ctenosaura hemilopha* resembles that of other *Ctenosaura* species, with simple, conical anterior teeth gradually progressing to tricuspid posterior teeth.

The five insectivorous taxa, *Anolis cristatellus*, *Basiliscus vittatus*, *Cordylus namakuiyus*, *Draco blanfordii*, and *Xenosaurus rectocollaris*, are from the Dactyloidae, Corytophanidae, Cordylidae, Agamidae, and Xenosauridae families, respectively. Although each specimen is from a different family, the tooth complexities are generally similar. The species average OPCR-values range between 5.431 and 9.865 PPT. Results follow that of previous work, with insectivores generally having higher complexities than carnivores, lower complexities than herbivores, and overlap with omnivore complexity levels [11]. Despite the relatively narrow range of tooth complexities, insectivores display a wide range of dental morphologies. *Anolis cristatellus* and *Basiliscus vittatus* show species average OPCR-values of 8.629 and 9.865 PPT, respectively, and display dentitions similar to many herbivorous species, with tricuspid teeth posteriorly that gradually progress to small, conical teeth anteriorly. *Draco blanfordii* (average OPCR = 8.977 PPT) has a similar complexity level to *A*. *cristatellus* and *B*. *vittatus*, but morphologically the teeth are much wider and the anterior teeth are significantly larger, sharper, and recurved. In contrast, the dentitions of *Cordylus namakuiyus* and *Xenosaurus rectocollaris* are much simpler (average OPCR = 6.873 and 5.431 PPT, respectively) and have blunt, rounded single-cusped teeth.

Three carnivorous species are included in the study: *Heloderma horridum*, *Shinisaurus crocodilurus*, and *Varanus prasinus*. Carnivores exhibit the lowest species average OPCR-values among all dietary categories, ranging between 4.098 and 7.716 PPT. The tooth morphologies of all three species are generally similar with entirely single-cusped, conical teeth. *Heloderma horridum* possesses long, narrow, sharp, re-curved teeth; *Shinisaurus crocodilurus* possesses slightly wider and blunter straight teeth; and *Varanus prasinus* possesses wide, sharp, recurved teeth that gradually increase in size posteriorly.

### Comparison of dental elements

**Fig 2:**
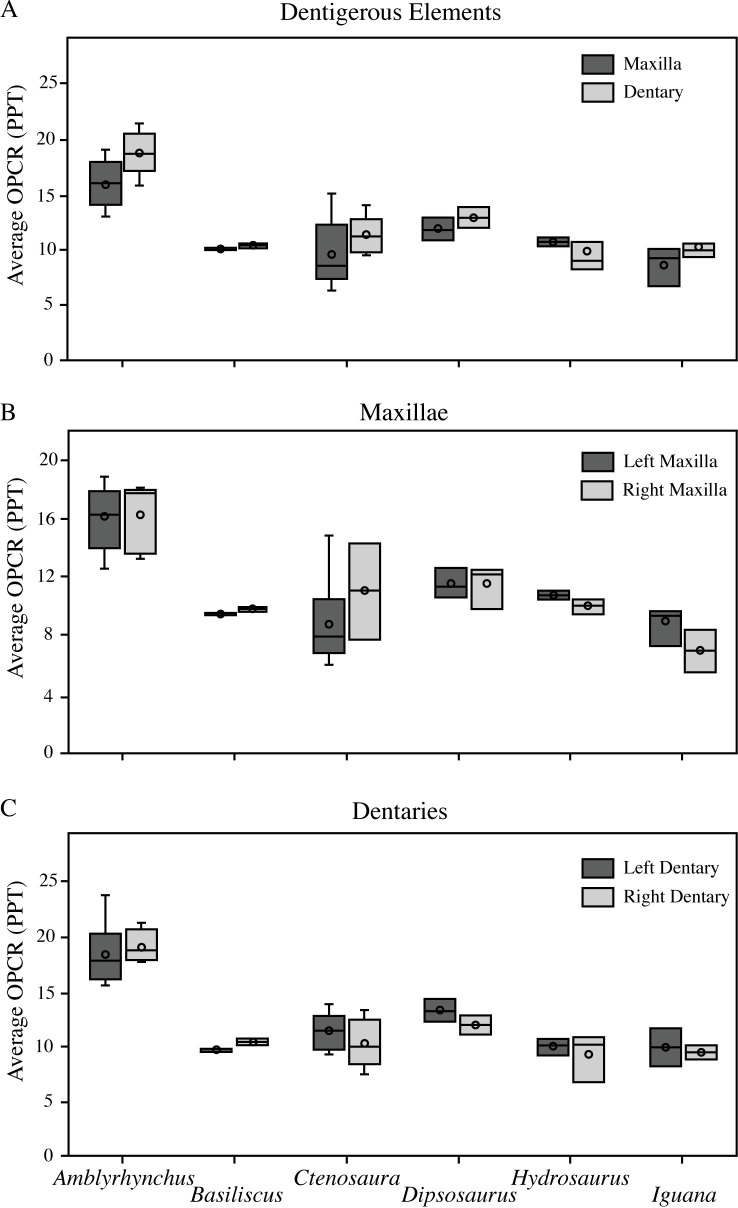
Observed range of dental complexity for six genera. A) Average OPCR of upper tooth row compared to lower tooth row; B) Average OPCR of left premaxilla and maxilla compared to right premaxilla and maxilla; C) Average OPCR of left dentary compared to right dentary. Average OPCR is calculated at 25 RPT and standardized for the number of teeth included in each element. Median is represented by a horizontal line, mean is represented by a circle, and range is represented by upper and lower short horizontal lines. Boxes encompass the exclusive median.

We examined the difference in complexity between the upper and lower tooth row, the left and right premaxilla and maxilla, and the left and right dentary (Fig 2). We observed minor complexity differences in each comparison, although the largest variation is between the upper and lower tooth row (Fig 2). The complexity of the dentary is consistently higher than the combined premaxilla and maxilla across all genera except *Hydrosaurus* (Fig 2A). Both the upper and lower teeth of the three *Hydrosaurus* species display simple, narrow, curved anterior teeth and stouter, blunter posterior teeth. In *Hydrosaurus amboinensis*, the posterior teeth are single-cusped in both the maxilla and dentary, but in *Hydrosaurus pustulatus*, the maxillary posterior teeth are distinct tricuspid teeth whereas the posterior teeth of the dentary are less defined tricuspid teeth. *Ctenosaura* does not show significant variation between upper and lower tooth row complexities (*P* = .12). The tooth morphology is very similar among *Ctenosaura* species, with simple, conical anterior teeth gradually progressing to tricuspid posterior teeth. The anterior teeth are simpler than the posterior teeth and there is no obvious difference in tooth morphology between the upper and lower tooth rows. There is a significant complexity difference between the upper and lower tooth rows of *Amblyrhynchus cristatus* (Fig 2; Table 3). Eight dentaries and eight premaxillae and maxillae were included in the comparison of *Amblyrhynchus* elements. Although the average OPCR of the dentary is notably higher than the premaxilla and maxilla (*P* = .03), the tooth morphology of the upper and lower tooth rows appear to be similar, consisting of tricuspid teeth with the same size and shape throughout the entire dentition.

**Table 3:** Comparisons of element complexity in *Ctenosaura* and *Amblyrhynchus* using average OPCR-values of each element.

Genus	Premaxilla and Maxilla vs. Dentary	Left vs. Right Premaxilla and Maxilla	Left vs. Right Dentary
*Ctenosaura*	0.12	–	0.26
*Amblyrhynchus*	**0.03**	0.89	0.64

A *P*-value of less than .05 is significant (bolded).

Across all genera, the left and right side of the upper tooth row and lower tooth row have very similar complexities; neither the left nor right side is consistently more complex than the other. The tooth morphology of the left and right dentigerous elements appears to be nearly identical in every specimen. Statistical tests for *Ctenosaura* and *Amblyrhynchus* do not show any significant differences between the left and right sides of the dentition (Table 3).

#### Complexity shifts through ontogeny

**Fig 3:**
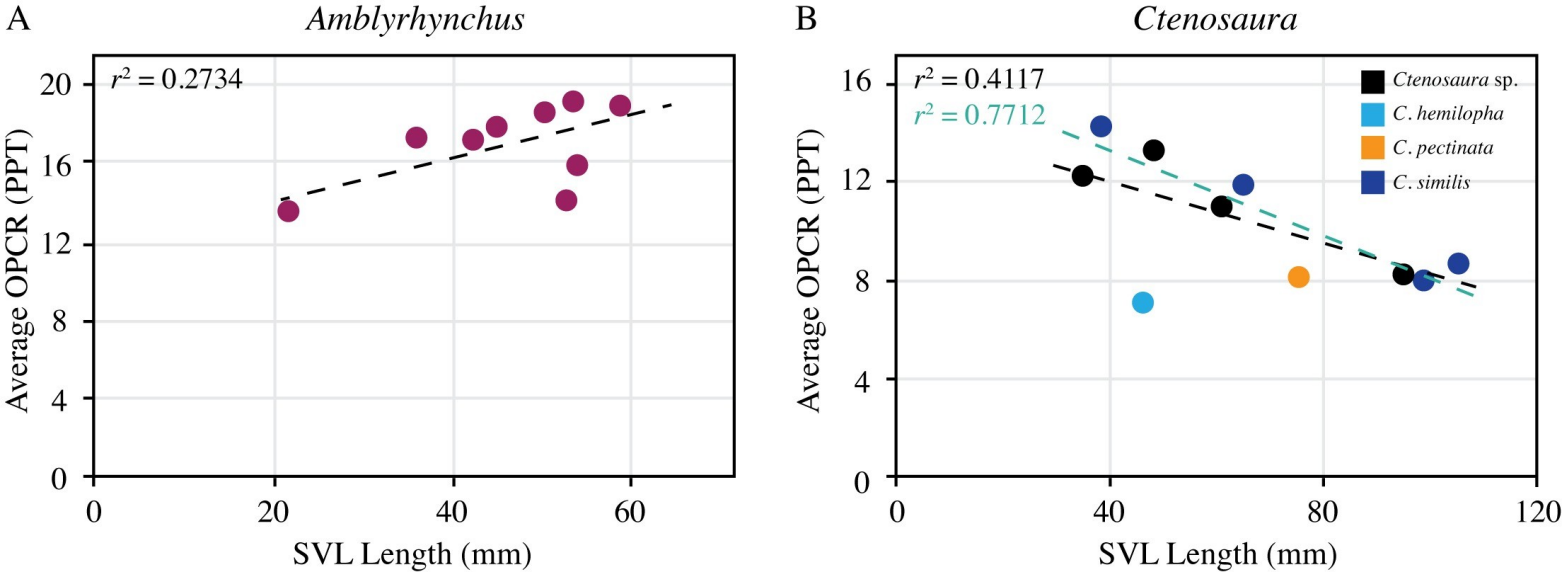
Trendlines displaying the relationship between skull length and dental complexity (avgOPCR) in two genera. A) The correlation between skull length and average OPCR for nine *Amblyrhynchus cristatus* specimens. B) The relationship between skull length and average OPCR for ten *Ctenosaura* specimens. The blue trend line includes all *Ctenosaura* species whereas the black trendline excludes *C*. *hemilopha* specimen.

Two genera, *Amblyrhynchus* and *Ctenosaura*, were assessed for a shift in dental complexity throughout their lifetimes. Other genera were not evaluated because there were too few specimens to accurately discern a trend. Nine *Amblyrhynchus cristatus* specimens were included in the ontogenetic comparison of dentition and diet. We found dental complexity grows larger as skull length increases. The individual with the shortest skull length (21.6 mm) has an average complexity of 13.486 PPT, whereas the individual with the longest skull length (58.9 mm) has a complexity of 18.78 PPT, with a gradation between these values (Fig 3). Although there is a visible correlation between skull length and dental complexity, the low *r*^2^ value of 0.27 suggests a less defined correlation between skull length and dental complexity.

Ten *Ctenosaura* specimens were included in the analysis of dental complexity shifts through ontogeny, including four *C*. *similis*, one *C*. *hemilopha*, one *C*. *pectinata*, and four *Ctenosaura* specimens without species identifications. One *Ctenosaura hemilopha* was not included because a skull length measurement was not available. Overall, as skull length increases, average OPCR-value decreases (i.e., older individuals possess simpler teeth) (Fig 3B). The *Ctenosaura* specimen with the shortest skull length of 34.7 mm has an average OPCR-value of 12.115 PPT. The species identification of this specimen is unknown, so it may be an insectivore or omnivore. The *Ctenosaura similis* specimen with the longest skull length of 105 mm has an average OPCR-value of 8.714 PPT. Average dental complexity values of *Ctenosaura* species range between 7.191 to 14.108 PPT. The *Ctenosaura hemilopha* specimen possesses the lowest dental complexity despite its relatively short skull length (46 mm) and is an outlier.

## Discussion

Our findings are exploratory and represent preliminary, but noteworthy, additions to the knowledge of squamate dental patterns. In particular, the statistical significance of some of our results, such as the ontogenetic trends, is limited by our sample sizes. In spite of these limitations, these data independently support previous hypotheses and also present intriguing trends not previously observed.

### Relationship between diet and dental complexity

Our results corroborate prior studies by showing a gradual increase in dental complexity from carnivores to herbivores [11]. This study recovers a range of complexities for herbivores (8.131–17.145 PPT), insectivores (5.431–9.865 PPT), and carnivores (4.098–7.716 PPT), which are similar to previous results [11]. The similarity between studies suggests that this quantitative method is repeatable and reliable when following similar procedures. These findings allow us to hypothesize the diet of extant and extinct taxa with increased precision using dental complexity and tooth morphology [9, 31–33].

Although each dietary category has a well-defined range of complexities, there is overlap between each group. The complexity range of insectivores and carnivores overlap considerably, and the Mann–Whitney *U* test reveals that these two groups do not have significantly different medians (*P* = 0.09). The complexity overlap may exist because both diets consist of over 90% animal material, only differing between the type of animal material consumed (vertebrates versus invertebrates). Additionally, the relatively small sample size may be playing a role in this result, as previous research recovered this overlap but it was not statistically significant [11]. Herbivores have significantly different complexities compared to both carnivores and insectivores (*P* < 0.01), but there is a wide range of complexities among this group. The range represents the different dental morphologies between species, however in rare cases OPCR-values may, in part, reflect analysis resolution. For example, adult *Amblyrhynchus cristatus* and *Iguana iguana* both consume plant material for nearly their whole diet, but the average OPCR of all *A*. *cristatus* individuals is 17.145 PPT whereas the average OPCR *I*. *iguana* individuals is 8.726 PPT. The high complexity value for *A*. *cristatus* is the result of complex tricuspid teeth throughout the entire dentition. In contrast, the relatively low complexity for *I*. *iguana* is a result of small tooth serrations, which results in these features not being detected, thus giving an artificially low complexity [11, 34]. Higher resolution analyses (40 and 50 RPT) may ameliorate this issue but also lead to problems directly comparing data from different sources [11].

### Element complexity variance

The complexity of the dentary is higher than the premaxilla and maxilla in all genera except *Hydrosaurus* (Fig 2). The low average dentary complexity in *Hydrosaurus* is likely because only the dentary of *H*. *amboinensis*, which has the lowest dental complexity (7.874 PPT) of the *Hydrosauru*s genus, was available for the analysis. The consistently higher dentary complexity across multiple taxa is unclear because there are no obvious differences in tooth morphology between the upper and lower teeth. Despite this pattern, the ranges consistently overlap (except in *B*. *vittatus* which only included one dentary that had a slightly lower OPCR-value than the sampled maxillae), and the mean values of the elements are very close. One exception to this trend is *A*. *cristatus*, in which the complexity of the dentary is significantly higher than the premaxilla and maxilla (*P* = .03), which is unexpected since both tooth rows consist of entirely tricuspid teeth. One possible explanation is that *A*. *cristatus* feeds by scraping algae off rocks which grinds down and preferentially flattens the occlusal surface of anterior premaxillary teeth [35], resulting in a lower complexity of the upper tooth row. Dental complexities of the left and right elements of both the upper and lower tooth rows are consistently similar and slight variation between elements may be the result of minor differential wear patterns (Fig 2).

Overall, these results demonstrate little difference in dental complexity between elements of an individual, regardless of species, which reflects similarities between the tooth morphologies of the upper and lower teeth. It remains unclear why the dentary is commonly more complex than the premaxilla and maxilla across multiple taxa, however, the differences in complexity are minute, not statistically significant, and may be a result of the small sample size. As expected, the left and right sides of the dentition are nearly identical, with slight variation most likely due to missing teeth or variance between individuals.

### Ontogenetic shift in dentition

We hypothesized that taxa that undergo a shift in diet also exhibit a shift in dental shape, and species that maintain a constant diet possess the same dental complexity throughout life. In particular, we expected *Ctenosaura* to demonstrate an increase in dental complexity as it matures and consumes more plant material and *Amblyrhynchus* to maintain a constant dental complexity because it is primarily herbivorous. However, the two genera assessed for an ontogenetic shift in dentition showed unexpected trends: *Ctenosaura* exhibited a decrease in dental complexity as skull length increased, whereas *Amblyrhynchus* showed an increase in dental complexity as skull length increased.

The *Ctenosaura* SVL to complexity correlation consisted of data from three species: *C*. *similis*, *C*. *pectinata*, and *C*. *hemilopha* (Fig 3). Of these species, *Ctenosaura similis* and *Ctenosaura pectinata* exhibit a shift in diet from insectivores as juveniles to herbivores as adults [13, 20, 24]. *Ctenosaura hemilopha* is classified as an omnivore and does not show the same shift in diet as the other two *Ctenosaura* species [36]. The *Ctenosaura hemilopha* specimen has a lower dental complexity (8.990 PPT) than expected for its measured skull length (46 mm) (Fig 3), which may be a reflection of its omnivorous diet. Another possible explanation for the low complexity of *C*. *hemilopha* is that it is missing several complex posterior teeth, which results in the average complexity being artificially low. Overall, we think the general decrease in tooth complexity in *Ctenosaura* from juvenile to adult may be partly explained by changes in tooth morphology. Juveniles possess small, straight, conical anterior teeth, whereas adults possess larger and posteriorly recurved anterior teeth. Adults also have a greater number of anterior teeth than juveniles [18]. The reason for the large, recurved anterior teeth is likely because adults occasionally feed on vertebrates, such as birds and juvenile lizards [37–39], so their large, recurved anterior teeth may function to aid in capturing these vertebrates. The posterior teeth of *Ctenosaura* juveniles and adults appear similar in morphology, although some adult specimens have blunter or less cuspate posterior teeth. Previous research by Torres-Carvajal [18] suggests that juvenile posterior teeth may have up to four or five cusps, but posterior adult teeth most commonly have only three cusps. Adults retain cuspate posterior teeth to process plant material because they rely increasingly on herbivory as they mature [13]. It has also been suggested that adults have a slower rate of posterior tooth replacement than anterior tooth replacement, which may cause their posterior teeth to become worn down [40]. Grinding plant material wears down the cusps and may result in lower overall complexity. Both the increase in size and number of anterior teeth in adults, as well as the slow replacement of posterior teeth likely account for the general decrease in complexity across *Ctenosaura*.

**Fig 4:**
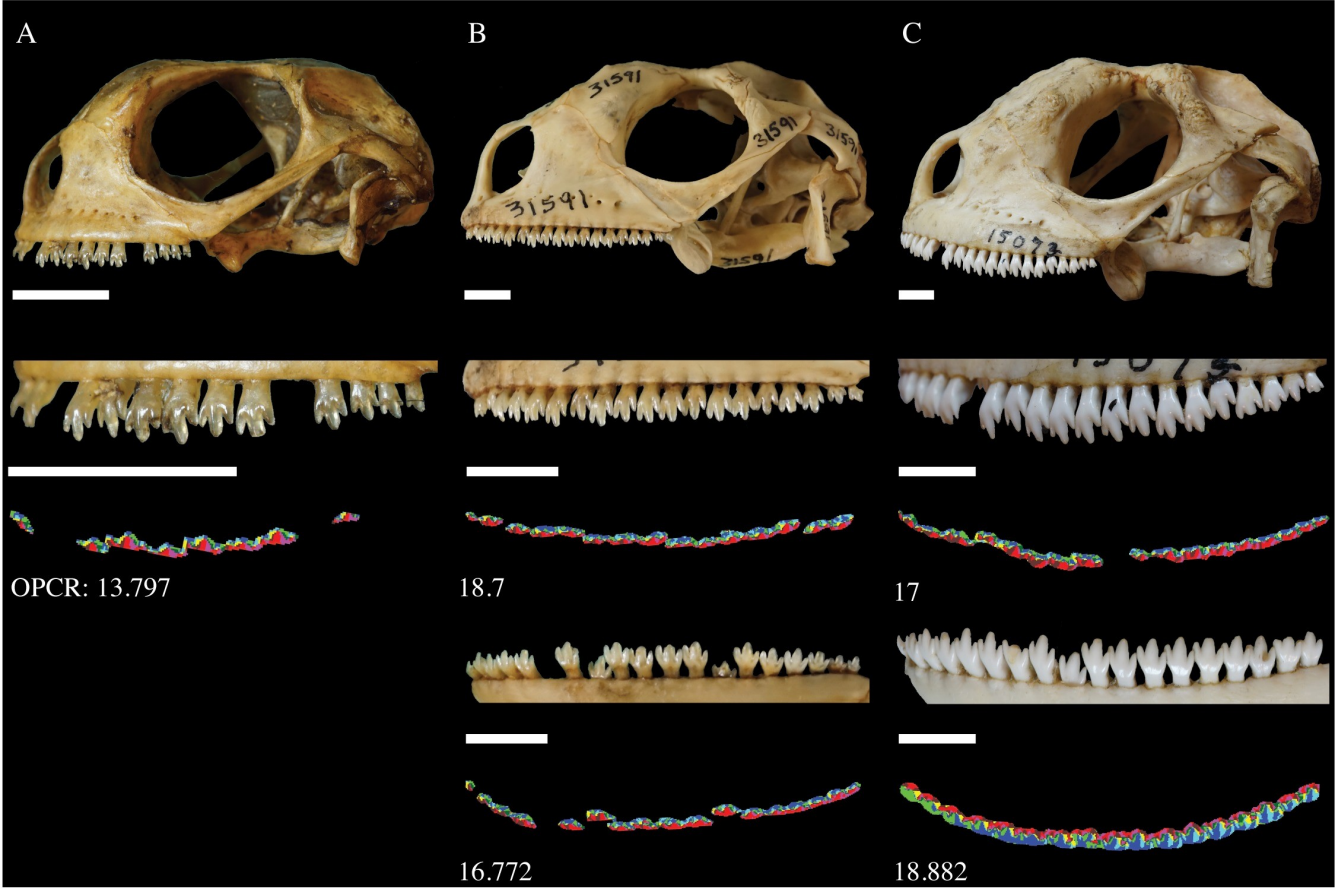
Dentigerous elements and OPCR maps of three *Amblyrhynchus cristatus* specimens with increasing skull length. A) Specimen FMNH 22100 with 21.6 mm SVL; B) Specimen AMNH 31591 with 45 mm SVL; C) Specimen FMNH 15073 with 58.9 mm SVL. Increasing SVL generally corresponds to larger teeth with more defined cusps. Scale bar equals 5 mm. OPCR maps show the occlusal view of the left upper tooth row and lower tooth row.

We attribute the gradual increase in dental complexity of *Amblyrhynchus cristatus* to changes in tooth size and cusp definition. Teeth with three cusps characterize both juvenile and adult individuals (Fig 4), which are used to scrape marine algae off rocks during feeding [15, 35]. Despite similarities in general morphology, juvenile teeth are smaller and overlap to a lesser degree than adult dentitions (Fig 4A). Furthermore, anterior teeth of juveniles are much smaller and simpler than posterior teeth, either having lateral cusps that are less-defined or absent. Adults possess larger and more defined tricuspid teeth both anteriorly and posteriorly, although the anterior teeth are still smaller and flatter across the occlusal surface compared to posterior teeth. The higher complexity of adult individuals appears to be a reflection of the increased tooth size and better-defined cusps. Given that adults exclusively consume algae, complex dentitions would allow for a more efficient breakdown during consumption. Previous research by Nagy and Shoemaker [15] indicates that juveniles possess a greater amount of animal matter (11%) in their diet, which typically corresponds to simpler dentitions [6, 11]. Therefore, animal consumption by *Amblyrhynchus* juveniles may also partially explain their lower dental complexity. The shift in dentition from simple to more complex matches the ontogenetic shift in diet from insectivores to herbivores and supports that dental complexity is strongly influenced by diet.

Although *Amblyrhynchus* adults exhibit more complex teeth than juveniles, older individuals have varying complexities depending on the amount of tooth wear. In adult specimens with low complexities, the central cusp is often worn down and relatively flat across the occlusal surface, which is likely from scraping algae off rocks. In contrast, adults with higher complexities have rounded and well-defined cusps which appear to be unworn. Previous studies suggest that *Amblyrhynchus cristatus* replace their teeth at higher rates than many other iguanids [41] and nearly all sampled adult specimens have replacement teeth emerging lingually in both the upper and lower tooth rows. The emerging teeth all have rounded, well-defined cusps, suggesting that the flat occlusal surface is a result of wear on teeth from feeding. It is likely that the high rate of tooth replacement allows *Amblyrhynchus* to retain high complexity at old ages despite the constant scraping and wearing down of their teeth.

### Hypothesizing diet using dental complexity

Two species included in the study have diets that are not well-documented in wild individuals. Using the calculated ranges of dental complexities and observations of tooth morphology, we are able to propose possible dietary categories for *Hydrosaurus amboinensis* and *Lanthanotus borneensis*. The specimen of *H*. *amboinensis* has an avgOPCR of 7.874 PPT which exists within the complexity ranges of insectivores (5.431–9.865 PPT) and omnivores (8.990 PPT). The dental morphology of *H*. *amboinensis* consists of sharp, conical, recurved anterior teeth and labiolingually-compressed, triangular-shaped posterior teeth. Similar dental morphologies exist among both insectivores and omnivores, so it is plausible that *H*. *amboinensis* could belong to either category. This species has a lower complexity (avgOPCR = 7.874 PPT) compared to the other two studied *Hydrosaurus* species (*Hydrosaurus pustulatus* = 10.502 PPT, *Hydrosaurus sp*. = 10.132 PPT), which falls outside of individual variation, suggesting that its diet may differ. *Hydrosaurus pustulatus* is entirely folivorous, but phylogenetic studies reconstruct an omnivorous Agamidae ancestor [24, 42, 43]. Given an ancestral omnivorous diet, it is possible that *H*. *amboinensis* could have retained omnivory instead of evolving herbivory like *H*. *pustulatus*. Taking dental complexity, tooth morphology, and evolutionary history into consideration, we hypothesize that *Hydrosaurus amboinensis* is likely an omnivore, consuming less plant material than its close relative.

Similarly, the diet of *Lanthanotus borneensis* is largely unknown, with relatively little research in general on wild individuals [44]. Document diet information relies on acceptance or rejection of food items fed in captivity, which reveals that this taxon eats prey such as squid, fish, and earthworms, but refuses various other animal material such as frog legs and mussel feet [44]. There is no information on whether it accepts or refuses plant material. Our quantitative analysis demonstrates that this species has a very low average dental complexity (avgOPCR = 4.232 PPT) that falls in the range of carnivore complexities (4.098–7.716 PPT). Previously measured squamate carnivores, such as some varanids and *Heloderma* also possess teeth with comparable complexities [11]. Furthermore, the specimen shows simple tooth morphology with long, recurved, sharp conical teeth throughout its entire dentition. Given the simple dental complexity and morphology, as well as documented animal prey this species ate while in captivity, it is likely that *L*. *borneensis* is a carnivore.

Overall, this study identifies several specific patterns in squamate dentition and expands previous quantitative work on the relationship between diet and dentition. Our data include additional taxa that support previous quantitative research in showing that dental complexity increases as squamates consume a greater amount of plant material [11]. Our findings also suggest that squamate dentition is nearly symmetrical in studied dentigerous elements (i.e., upper tooth row and lower tooth row). This study demonstrates that sampled upper and lower teeth have similar complexities and are nearly identical on the left and right sides of the mouth. Although broader conclusions are limited by our small sample size, these preliminary results demonstrate intriguing patterns through ontogeny in sampled squamates. We found that *Ctenosaura* and *Amblyrhynchus* undergo opposite shifts in complexities through ontogeny. *Ctenosaura* complexity decreases from juvenile to adult, possibly because adults increase size and number of simple anterior teeth that aid in occasional capture of vertebrates. *Amblyrhynchus* complexity increases through ontogeny, which may be due to teeth becoming larger and cusps becoming more defined. Ontogenetic shift in diet appears to influence the shift in dentition, although complexity seems to shift based on species-specific changes in tooth morphology, rather than shifting to a complexity associated with the new adult diet as we hypothesized.
